# A Protocol for Measurement of Noncoding RNA in Human Serum

**DOI:** 10.1155/2012/168368

**Published:** 2012-07-01

**Authors:** Caroline J. Taylor, Sarang N. Satoor, Amaresh K. Ranjan, Maria V. Pereira e Cotta, Mugdha V. Joglekar

**Affiliations:** ^1^O'Brien Institute, 42 Fitzroy Street, Fitzroy, VIC 3065, Australia; ^2^Department of Surgery, St. Vincent's Hospital, University of Melbourne, Melbourne, VIC, Australia; ^3^Faculty of Health Sciences, The Australian Catholic University, VIC, Australia; ^4^DNA Sequencing Facility, Microbial Collection Center and National Center for Cell Science, Ganeshkhind Road, Pune MH 411007, India; ^5^Cardiovascular Research Institute, Mount Sinai School of Medicine, 1 Gustave L. Levy Place, New York, NY 10029-6574, USA; ^6^NHMRC Clinical Trials Centre, The University of Sydney, Rm 316, Level 1, Medical Foundation Building, 92 Parramatta Road, Camperdown, NSW 2050, Australia

## Abstract

MicroRNAs (miRNAs) are small noncoding RNAs that act as regulators of gene expression by targeting mature messenger RNAs. Following the initial report of the presence of miRNAs in serum and plasma a number of studies have successfully demonstrated the use of these miRNAs as biomarkers of disease. Currently, there are many methods of isolating total RNA from liquid samples. Here, we describe a simple, cost effective method for extraction of RNA from human serum as well as subsequent real time PCR analysis of miRNA levels.

## 1. Introduction

MicroRNAs (miRNAs) are small noncoding RNAs that act as regulators of gene expression by targeting mature messenger RNAs (mRNAs). Recently, miRNA has been discovered in both serum and plasma, where it appears to be relatively stable [1]. Since this initial report, there have been a number of studies examining miRNA expression in either serum or plasma in diseases including various cancers [2], cardiovascular disease [3], and myopathies such as Duchenne muscular dystrophy [4]. The methods of miRNA isolation, as well as the techniques used to examine expression, vary in these studies. Here, we provide a protocol for the isolation of RNA from serum and subsequent determination of miRNA expression levels using TaqMan-based real-time PCR detection. This procedure, from isolation of RNA to acquisition of real-time PCR data, takes around 8 hours. 

## 2. Materials

### 2.1. Reagents

The materials used are as follows:

 Chloroform (Labserv cat # CL728).
 Ethanol (Labserv cat # BSPE6975); *prepare 75% ethanol (v/v) by diluting 100% ethanol in nuclease-free water; we recommend making fresh ethanol solution every time the protocol is carried out. *
 Glycogen (molecular biology grade) (Sigma, cat # G1767) (20 *μ*g/*μ*L).
 Nuclease-free water (Qiagen, cat # 129117).
 Isopropanol.
 Taqman Fast Universal Master Mix (2x) (Life Technologies cat #4366073).
 Taqman microRNA assays (Life Technologies, various).
 
*The miRNA-specific RT primers and TaqMan probe-primer mix are supplied together as an individual assay for a single miRNA*.
 TaqMan microRNAs reverse transcription *kit (Life Technologies 4366597)*.
 
*Kit includes 10X RT buffer, *100 mM* dNTP mix, RNase inhibitor (*20 U/*μ*L*), and multiscribe RT enzyme *(50 U/*μ*L)*; the enzyme must be kept at −20*°*C at all times.*
 TriReagent (Ambion AM9738).

### 2.2. Equipment

 Optical Adhesive film for PCR (Applied Biosystems, cat # 4311971).
 Centrifuge, refrigerated (with rotor for 1.5 mL tubes, e.g., Heraeus Fresco).
 Centrifuge, refrigerated (with rotor for 96-well plates and 15 mL tubes, e.g., Heraeus Multifuge X1).
 Ice.
 Micropipettor and filter tips.
 Microplates (96-well half skirt, Axygen Cat # PCR-96-M2-HS-C).
 MicroAmp Fast Optical 96-well reaction plate (Applied Biosystems cat #4346906).
 Real-time PCR system (7900 HT Fast Real-Time PCR system; Applied Biosystems Cat # 4351405).
  Tubes, microcentrifuge (1.5 mL and 2 mL).
 Vortex mixer.
 8 mL serum separator clot activator vacuette (Greiner Cat # 455078).
 21-gauge needle push button blood collection sets (BD Cat # 367344).

## 3. Method

### 3.1. Sample Collection

It is important that samples are collected in accordance with the appropriate ethical guidelines.

1. Collect blood samples using 21-gauge needle push button blood collection sets into 8 mL serum separator clot activator vacuettes.
2. Invert the tubes five times before allowing clotting for 30 minutes.
3. Centrifuge the tubes for 10 minutes at 1,000–1,300 g in a swinging bucket rotor.
4. Remove serum from the tube and aliquot into 500 *μ*L volumes in 1.5 mL microcentrifuge tubes.

Serum can be used immediately or can be frozen at −80°C for future use.

### 3.2. RNA Isolation

(5) Transfer 400 *μ*L of serum into a 2 mL microcentrifuge tube.
(6) Add 1 mL TRI reagent to each tube. *Following addition of TRI reagent to the serum, yellow globules appear in the solution. These dissipate following vortexing and incubation and do not appear to interfere with downstream processing*.
(7) Add 1 *μ*L of (1 *μ*g/uL) nuclease-free glycogen to each tube. Vortex for 20 seconds. Incubate at room temperature for 10 minutes
(8) Add 200 *μ*L of chloroform to each tube and shake vigorously for 20 seconds. *This ensures proper mixing of the chloroform and TRI reagent immediately after addition. Tubes should not be vortexed at this stage. Due to the time involved in each stage of processing we recommend that a maximum of 12 tubes be processed at a time. *
(9) Incubate at room temperature for 15 minutes
(10) Centrifuge at 12,000 g for 15 minutes at 4°C
(11) Transfer 800 *μ*L of the upper aqueous phase to a fresh 2 mL microcentrifuge tube. *There will be a large amount of white material at the interface of the aqueous and organic layers. Following removal of 800 μL from the aqueous layer, there will be residual aqueous phase remaining. *
(12) Add 1.2 mL isopropanol to each tube as 2 × 600 *μ*L volumes. Vortex for 5 seconds.* At this stage the tube will be very full. *
(13) Incubate at room temperature for 10 minutes.
(14) Centrifuge at 12,000 g for 8 minutes at 4°C. *Whilst a pellet should be visible, it is still important to orient the tubes before centrifugation so that the hinge of the lid faces the outer rim of the centrifuge. *
(15) Carefully aspirate the supernatant by placing the pipette tip along the wall of the microcentrifuge tube opposite from the hinge (and therefore the pellet). *Due to the large volume of liquid needing to be removed it may be possible to carefully tip out the contents of the tube. *
(16) Add 1 mL 75% ethanol to each tube and invert the tube 5 times
(17) Centrifuge at 7,500 g for 5 minutes at room temperature. Again, place the tubes in the same orientation inside the centrifuge.
(18) Carefully remove the supernatant as described in step (11). In order to ensure that as much of the ethanol is removed as possible it may be necessary to sequentially decrease the size of the pipette tip used.
(19) Allow the tubes to dry out for 5 minutes at room temperature.
(20) Add 20 *μ*L of nuclease-free water to each tube and resuspend pellet thoroughly.* Always store the RNA on ice after this stage*.
(21) Measure the concentration of total RNA using a NanoDrop ND-1000. *We have observed that the 260/280 ratio is often around 1.3. This does not appear to have an effect on downstream processing or data generated. *

RNA obtained using the above protocol is used for miRNA-specific reverse transcription. Remaining RNA can be stored at −80°C.

### 3.3. miRNA-Specific Reverse Transcription

For ease of handling, we recommend that reverse transcription is carried out in either 8-well 0.2 mL strip tubes or a 96-well thin walled PCR plate.

(22) 10 ng of RNA is reverse transcribed for each miRNA primer. Calculate the volume of RNA required to provide 10 ng final concentration per primer adding an extra reaction in case of error. Add nuclease-free water to make the volume up to 1.67 *μ*L per tube.
(23) Thaw 10x RT buffer, RNase inhibitor, and 100 nM dNTPs (provided in TaqMan microRNAs Reverse Transcription Kit) on ice. *The multiscribe reverse transcriptase must remain at −20 *°*C until required. *
(24) Prepare master mix by adding the above reagents as per the calculations shown in Table 1. Calculations are shown for each miRNA and 12 serum samples. Total reaction volume is 5 *μ*L. An error of 0.5 reactions is incorporated into the calculations. *Master mix must be mixed by trituration and should remain on ice at all times. *
(25) Add 2.33 *μ*L of RT master mix and 1 *μ*L of miRNA-specific primer to each well, then add 1.67 *μ*L of RNA to all wells. All additions must be made on ice.
(26) Vortex and spin down so that the RNA and master mix are thoroughly mixed.
(27) Reverse transcription is carried out using a BioRad DNA engine with the following program
(1) 16°C for 30 min
(2) 42°C for 30 min
(3) 85°C for 5 min
(4) 4°C on hold.

### 3.4. Real-Time PCR for miRNAs

(28) Thaw the assay-on-demand (AOD) on ice. Prepare the master mix for each target miRNA to be detected as per the calculations shown in Table 2. These calculations are based on 12 samples and have an error of 1 reaction included in the calculation. Total reaction volume is 5 *μ*L/well.
(29) For each target miRNA, add 4.2 *μ*L of the specific master mix to each well of a 0.1 mL optically clear PCR plate, followed by 0.8 *μ*L of the cDNA prepared using miR-specific RT primers in step (27). Cover the plate with an optically clear film. *It is important to ensure that the wells around the edge of the plate are fully sealed as they are more susceptible to evaporation. *
(30) Centrifuge the plate at 3,500 rpm for 5 minutes to ensure that all contents are at the bottom of the well and all air bubbles have been eliminated.
(31) Place the plate in a 7900 HT fast real-time PCR machine and run it in fast mode using the following program
Stage  1 : 95°C for 20 sec
Stage  2 : 40 cycles of
     95°C for 1 sec,
     60°C for 20 sec.

**Figure 1:**
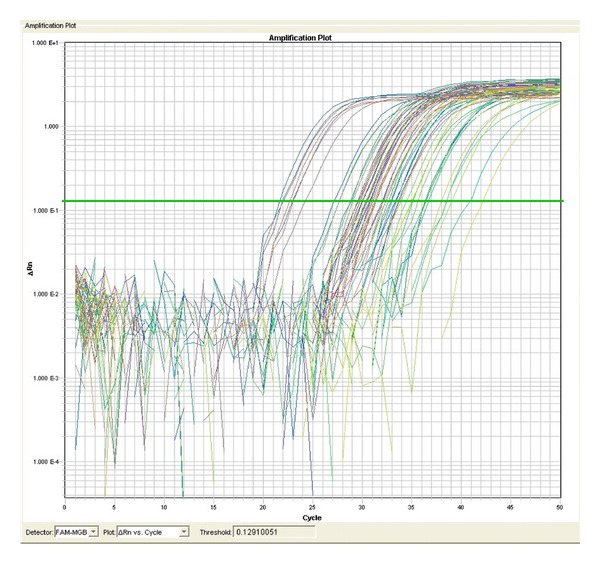
miRNA levels in human serum. RNA was isolated and PCR carried out as described here. Data show the expression of 8 miRNAs from 8 human serum samples.

**Figure 2:**
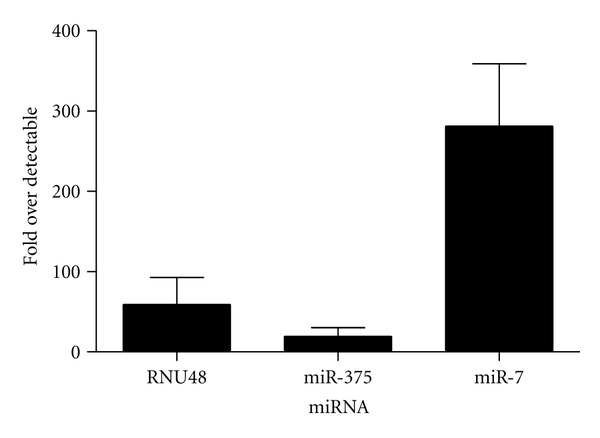
Representative real-time PCR data obtained from human serum. Six human serum samples were processed using the protocol described above and expression of miR-375 and miR-7 as well as the control miRNA RNU48 was examined. Data is expressed as fold over detectable.

The plate is read during the extension step of stage 2. An example of data obtained is shown in Figures 1 and 2.

## Figures and Tables

**Table 1:** 

	*μ*L/reaction	No. of reactions	Volume to add (*μ*L)

10X RT buffer	0.5	12	6.25
100 nM dNTPs	0.05	12	0.625
RNase inhibitor	0.03	12	0.375
Nuclease-free water	1.42	12	17.75
Reverse transcriptase	0.33	12	4.125

Total volume (*μ*L)	2.33		

**Table 2:** 

	*μ*L/reaction	No. of reactions	Volume to add
2X Fast PCR mastermix	2.5	12	32.5
20X AOD	0.25	12	3.25
Nuclease-free water	1.45	12	18.85

Total volume	4.2		
